# Supplementary material for: Mapping ActEarly: Using a child health map to evaluate a City Collaboratory programme on early promotion of good health and wellbeing
Source: PLoS One. 2026 Jan 16;21(1):e0326825. doi: 10.1371/journal.pone.0326825 (PMC12810778; doi:10.1371/journal.pone.0326825)
Supplement: S1 Table — (DOCX) [file pone.0326825.s001.docx]

**Table S1 ActEarly Project list**

| **Project number** | **Project title** | **Start date** | **End date** |
| --- | --- | --- | --- |
| 1 | The effectiveness evaluation of the [Join Us: Move Play (JU:MP) programme](https://www.activebradford.com/jump) | Jul-21 | Jul-25 |
| 2 | Whole system physical activity strategy: A process evaluation of the strategic and neighbourhood design delivery and evaluation of JU:MP | Mar-20 | Mar-25 |
| 3 | Understanding how to develop young leaders and evaluating [JU:MP Leads](https://www.activebradford.com/ju-mp-leads-programme) | Apr-21 | Mar-24 |
| 4 | Children and families process evaluation of JU:MP | Sep-21 | Mar-24 |
| 5 | Faith Settings Living Well (this includes Active Faith Settings and obesity trailblazer) | Jan-20 | Apr-24 |
| 6 | JU:MP@Home | May-20 | Jul-22 |
| 7 | JU:MP organisational evaluation including organisational change and individual project monitoring (JUMP) | Sep-21 | Sep-24 |
| 8 | [Creating Active Schools](http://www.creatingactiveschools.org/) evaluation (JUMP) | Sep-21 | Mar-24 |
| 9 | Green space development and evaluation (JU:MP) | May-22 | Sep-24 |
| 10 | [Play in Urban Spaces for Health](https://www.ucl.ac.uk/ioe/departments-and-centres/thomas-coram-research-unit/research/children-young-people-and-families/play-urban-spaces-health-push) (PUSH, formally Urban Forest Schools). | Mar-23 | Jul-24 |
| 11 | [BiB Breathes](https://borninbradford.nhs.uk/what-we-do/studies/bib-breathes/) | Jul-20 | Jun-26 |
| 12 | Healthy School Streets in TH and Bradford | Mar-20 | Jun-24 |
| 13 | Evaluation of Health Impact Assessment (HIA) policy in TH | Apr-20 | Dec-23 |
| 14 | [Measuring the built environment in studies of child health – a meta-narrative review of associations](https://www.mdpi.com/1660-4601/18/20/10741) | Jun-20 | Oct-21 |
| 15 | [ATHLETE](https://athleteproject.eu/): ATHLETE (Advancing Tools for Human Early Lifecourse Exposome Research and Translation) | Jan-21 | Jan-23 |
| 16 | [Systematic review of street-scale interventions and child health](https://www.mdpi.com/1660-4601/19/9/5227) | Nov-21 | Apr-22 |
| 17 | [Synergies between housing and local environments for enhanced child wellbeing in Tower Hamlets and Bradford (pilot study)](https://www.mdpi.com/1660-4601/19/19/12563) | Jun-21 | Sep-22 |
| 18 | Fast-food Exposure and Childhood Obesity in Tower Hamlets and Bradford | Nov-22 | Aug-23 |
| 19 | Healthy Environment Indicators for Bradford | May-23 | May-24 |
| 20 | [Food Improvement Goals in Schools (FIGS)](https://www.qmul.ac.uk/ceg/media/ceg/documents/FIGS-Conference-Project-Walk-poster.pdf) | Sep-22 | Aug-24 |
| 21 | [Fix our Food](https://www.foodsecurity.ac.uk/research/foodsystems-spf/) | Jan-21 | Dec-25 |
| 22 | [Ethnic cut-offs for defining overweight and obesity status using NCMP data](https://academic.oup.com/jpubhealth/article/42/4/e541/5706873?searchresult=1) | Oct-18 | Sep-23 |
| 23 | Using child measurement to better support parents, children & families with excess weight | Dec-21 | Unknown |
| 24 | Free School Meals Quantitative Evaluation in Primary School (HDRC) | Nov-22 | 2025 |
| 25 | [CONNECTS-Food project: Whole school approaches to food](https://www.york.ac.uk/healthsciences/research/public-health/projects/connects-food/connects-about/) | 2021 | Jul-22 |
| 26 | Community food assets evaluation | 2020 | Aug-25 |
| 27 | Yorkshire based evaluation Citizen science project asking young people in secondary schools to test out options and selection based on FSM allowance | Mar-23 | Nov-23 |
| 28 | [Development of the Centre for Coproduction and Peer Research (COPPER)](https://borninbradford.nhs.uk/about/our-funders/creating-energetic-and-sustainable-community-research-partnerships-developing-the-co-production-and-peer-research-copper-network-to-improve-health-and-reduce-inequality/) | Sep-23 | Apr-25 |
| 29 | Evaluation of the ActEarly Coproduction activities | Sep-23 | Aug-24 |
| 30 | Food consumption pattern and nutrient intakes in children living in Tower Hamlets (Action on Salt and Sugar Project) | Sep-22 | Sep-25 |
| 31 | Impact of a community alliance welfare benefits advice programme co-located in primary care: a mixed methods uncontrolled before and after study | Sep-21 | Unknown |
| 32 | Earning and Learning: Work-care decisions and young children in Tower Hamlets | Jan-22 | Sep-22 |
| 33 | Using the Urban iBox with children in Tower Hamlets (TH) and Bradford | Mar-22 | Sep-22 |
| 34 | Healthy Wealthier Families in East London (formerly *Colocation of welfare benefits related to maternity care)* | Mar-23 | Jun-24 |
| 35 | Inequalities of Access to Early Childhood Provision: the case of Early Learning at 2 in Tower Hamlets | May-22 | Apr-23 |
| 36 | Inequalities of access to Early Years Care and Education in Tower Hamlets and Bradford: mapping, focus groups and interviews | Feb-23 | Aug-24 |
| 37 | [Electronic Development Support Tool (EDST) (Previously "DATA 1"/"ASD Data Linkage")](https://caer.org.uk/data-1/) | 2021 | Unknown |
| 38 | [Glasses in Classes](https://caer.org.uk/glasses-in-classes/) | Unknown | Unknown |
| 39 | ClassACT (Covid Air Disinfectant System) | Jan-21 | Unknown |
| 40 | [SUCCESS](https://caer.org.uk/success/) | 2019 | Unknown |
| 41 | [FUNMOVES](https://caer.org.uk/funmoves/) | 2018 | Unknown |
| 42 | [Digital Makers](https://caer.org.uk/digital-makers/) | Unknown | Unknown |
| 43 | School Staff Welfare Support Scheme | 2020 | Unknown |
| 44 | [CAER Ask the Expert Webinars](https://caer.org.uk/webinars/) | 2020 | Unknown |
| 45 | Evaluation of the BDCT Family Support Worker Pilot Programme | Apr-23 | Oct-23 |
| 46 | [Act Locally](https://www.bradfordbirthto19.co.uk/educational-strategy/ealc/act-locally-info) (previously Holmewood) | 2022 | Unknown |
| 47 | [APPG Child of the North](https://www.thenhsa.co.uk/app/uploads/2023/01/COTN-APPG.pdf) | Nov-22 | Unknown |
| 48 | Poverty Proofing the School Day | 2022 | Unknown |
| 49 | Exempt accommodation/supported housing | 2022 | Unknown |
| 50 | Antipoverty coordination group | Unknown | Unknown |
| 51 | [Universal Basic Income work](https://actearly.org.uk/wp-content/uploads/2021/05/FINAL-Universal-Basic-Income-briefing-09-05-2023.pdf) | 2019 | Unknown |
| 52 | Active Bradford Whole System Physical Activity Strategy | Nov-22 | Dec-23 |
| 53 | Bradford Food Strategy support and evaluation | Unknown | Unknown |
| 54 | Lived experience of household overcrowding to inform development of overcrowding metrics (Tower Hamlets + Islington) | Oct-23 | Sep-24 |
| 55 | [Realist review of interventions to mitigate the health effects of household overcrowding](https://www.crd.york.ac.uk/prospero/display_record.php?ID=CRD42023396754) | Jan-23 | Dec-23 |
| 56 | Co-Production-Open Space events in Tower Hamlets | Jan-21 | Apr-25 |
| 57 | Tower Hamlets COVID-19 Walk-In Testing Sites Location Planning | May-20 | Aug-20 |
| 58 | [UBI for young adults pilot design](https://cascadewales.org/research/the-welsh-basic-income-evaluation/) (Welsh gov basic income pilot) | Oct-22 | Oct-26 |
| 59 | [Understanding ethnic variations in the prevalence and experience of food insecurity, and its interaction with mental health: A mixed methods longitudinal research programme.](https://www.york.ac.uk/healthsciences/research/public-health/projects/ethnic-variations-and-food-insecurity/#tab-2) | Oct-20 | Oct-23 |
| 60 | Evaluation of Tower Hamlets Community Food Hub | Jan-20 | Jan-21 |
| 61 | Inequalities of access to early years services and facilities | Feb-23 | Aug-24 |
| 62 | [Unlocking data to inform public health policy and practice](https://pmc.ncbi.nlm.nih.gov/articles/PMC9682213/) | May-21 | Dec-21 |
| 63 | [BABi Network](https://www.bradfordresearch.nhs.uk/2023/07/03/national-ground-breaking-birth-cohort-study-reaches-20000-participants/) - e-birth cohort - consent platform for routine data | Jan-20 | Jan-22 |
| 64 | Supporting Young Bradford: Understanding the barriers and enablers of emotional support for young people | Sep-22 | May-25 |
| 65 | [HUP North](https://environment.leeds.ac.uk/dir-record/research-projects/2075/healthy-urban-places-a-systems-approach-to-understanding-how-to-harness-the-power-of-local-places-to-improve-population-health-and-reduce-inequalities-hup-north) | Apr-24 | Apr-28 |
| 66 | [Asset Mapping Visualisation](https://www.ucl.ac.uk/culture/projects/2020-beacon-bursaries-awarded) | Dec-20 | Dec-21 |
| 67 | [Family Playrooms](https://www.bbbc.org.uk/family-playrooms/) and Have Your Say work | Jan-20 | Unknown |
| 68 | [Local Authority Research System](https://actearly.org.uk/wp-content/uploads/2020/12/Appendix-D-Report-version-for-our-local-partners.pdf) | May-20 | Dec-20 |
