# Supplementary material for: Mapping ActEarly: Using a child health map to evaluate a City Collaboratory programme on early promotion of good health and wellbeing
Source: PLoS One. 2026 Jan 16;21(1):e0326825. doi: 10.1371/journal.pone.0326825 (PMC12810778; doi:10.1371/journal.pone.0326825)
Supplement: Attachment — Submitted filename: Response to Reviewers.docx [file pone.0326825.s005.docx]

Date: 16/11/2025

Response to reviewers,

Dear Editor(s)

Thank you for the time taken to consider this article for publication, and to the peer reviewers for their thoughtful comments. We have responded to all queries as outlined below.

With warm wishes

Patience Gansallo and co-authors

1. Please ensure that your manuscript meets PLOS ONE's style requirements, including those for file naming. The PLOS ONE style templates can be found at

[https://journals.plos.org/plosone/s/file?id=wjVg/PLOSOne_formatting_sample_main_body.pdf](https://eu-west-1.protection.sophos.com/?d=plos.org&u=aHR0cHM6Ly9qb3VybmFscy5wbG9zLm9yZy9wbG9zb25lL3MvZmlsZT9pZD13alZnL1BMT1NPbmVfZm9ybWF0dGluZ19zYW1wbGVfbWFpbl9ib2R5LnBkZg==&p=m&i=Njc5M2RiN2I3MDBmMzgwZGNiNjZlMmNj&t=WURJZWY4UlFqM0UwVVFlUXduVWMvSnVUcnFUWjgrN21aUUdtYXlQYmdtRT0=&h=46b15574669043d98f5ab3165efe708a&s=AVNPUEhUT0NFTkNSWVBUSVbCfIhBZmGikoRw8ABdCOMdNfn1iHAptTsT5eekIRjGwQ) and

[https://journals.plos.org/plosone/s/file?id=ba62/PLOSOne_formatting_sample_title_authors_affiliations.pdf](https://eu-west-1.protection.sophos.com/?d=editorialmanager.com&u=aHR0cHM6Ly90cmFjay5lZGl0b3JpYWxtYW5hZ2VyLmNvbS9DTDAvaHR0cHM6JTJGJTJGam91cm5hbHMucGxvcy5vcmclMkZwbG9zb25lJTJGcyUyRmZpbGUlM0ZpZD1iYTYyJTJGUExPU09uZV9mb3JtYXR0aW5nX3NhbXBsZV90aXRsZV9hdXRob3JzX2FmZmlsaWF0aW9ucy5wZGYvMS8wMTBmMDE5YTA3NDg0ZmU4LTE0N2JjZTI0LTdkZWQtNGQwMS1iYjliLThmNDFkYTExMjRjZC0wMDAwMDAvdnJkU05IZGpNbDJuNmEyVFdwMllLX3VLUmc0cEFLcEFSdXdYOEQ2bGVucz0yMzQ=&p=m&i=Njc5M2RiN2I3MDBmMzgwZGNiNjZlMmNj&t=T0RhOXZEbXduMEFzWW5OMEMxTjBpc0xjaU12UjFLWWhSdFlnOWczRnBORT0=&h=46b15574669043d98f5ab3165efe708a&s=AVNPUEhUT0NFTkNSWVBUSVbCfIhBZmGikoRw8ABdCOMdNfn1iHAptTsT5eekIRjGwQ).

**Thank you, we have amended the formatting of the article. All figures have been checked.**

2. As you did not seek ethical oversight for the current synthesis of existing reports, please could you remove all ethical approval documents from your submission.

**Ethical approval documents were not included in this submission.**

3. Thank you for stating the following financial disclosure:

“Authors MB, JS and SI received a UK Prevention Research Partnership (MR/S037527/1) award for this study/project, which is funded by the British Heart Foundation, Cancer Research UK, Chief Scientist Office of the Scottish Government Health and Social Care Directorates, Engineering and Physical Sciences Research Council, Economic and
Social Research Council, Health and Social Care Research and Development Division
(Welsh Government), Medical Research Council, National Institute for Health
Research, Natural Environment Research Council, Public Health Agency (Northern Ireland), The Health Foundation and Wellcome.”

Please state what role the funders took in the study.  If the funders had no role, please state: "The funders had no role in study design, data collection and analysis, decision to publish, or preparation of the manuscript."

If this statement is not correct you must amend it as needed.

Please include this amended Role of Funder statement in your cover letter; we will change the online submission form on your behalf.

**Our role of funder statement reads:**

**“The funders had no role in study design, data collection and analysis, decision to publish, or preparation of the manuscript”.**

4. Thank you for stating the following in your manuscript:

“This work was supported by the UK Prevention Research Partnership (MR/S037527/1).”

We note that you have provided additional information within the Acknowledgements Section that is not currently declared in your Funding Statement. Please note that funding information should not appear in the Acknowledgments section or other areas of your manuscript. We will only publish funding information present in the Funding Statement section of the online submission form.

Please remove any funding-related text from the manuscript and let us know how you would like to update your Funding Statement. Currently, your Funding Statement reads as follows:

“Authors MB, JS and SI received a UK Prevention Research Partnership (MR/S037527/1) award for this study/project, which is funded by the British Heart Foundation, Cancer Research UK, Chief Scientist Office of the Scottish Government Health and Social Care Directorates, Engineering and Physical Sciences Research Council, Economic and Social Research Council, Health and Social Care Research and Development Division (Welsh Government), Medical Research Council, National Institute for Health Research, Natural Environment Research Council, Public Health Agency (Northern Ireland), The Health Foundation and Wellcome.”

Please include your amended statements within your cover letter; we will change the online submission form on your behalf.

**The additional information on funding has been removed from the manuscript. We have amended the funding statement below:**

**“Authors MB, JS and SI received a UK Prevention Research Partnership (MR/S037527/1) award for this study/project, which is funded by the British Heart Foundation, Cancer Research UK, Chief Scientist Office of the Scottish Government Health and Social Care Directorates, Engineering and Physical Sciences Research Council, Economic and Social Research Council, Health and Social Care Research and Development Division (Welsh Government), Medical Research Council, National Institute for Health Research, Natural Environment Research Council, Public Health Agency (Northern Ireland), The Health Foundation and Wellcome.”**

**“The funders had no role in study design, data collection and analysis, decision to publish, or preparation of the manuscript”.**

5. If the reviewer comments include a recommendation to cite specific previously published works, please review and evaluate these publications to determine whether they are relevant and should be cited. There is no requirement to cite these works unless the editor has indicated otherwise.

**We have not been asked to cite any specific previously published work**.

6. Please review your reference list to ensure that it is complete and correct. If you have cited papers that have been retracted, please include the rationale for doing so in the manuscript text or remove these references and replace them with relevant current references. Any changes to the reference list should be mentioned in the rebuttal letter that accompanies your revised manuscript. If you need to cite a retracted article, indicate the article’s retracted status in the References list and also include a citation and full reference for the retraction notice.

**We have not retracted any previously cited paper but have included few references to elaborate our responses to the reviewers. The reference list has been reviewed and is complete and correct.**

Additional Editor Comments (if provided)

Dear Authors,

Thank you for your submission which will make a great contribution to the literature. Both reviewers found this paper well developed and written. They have made several suggestions to further strengthen it which I ask you take on board. In addition, please address the following:
- It would be helpful to have a little more detail on the methods: whose responsibility was it to update the ActEarly project Log and how? Given how many projects, were there much missing data and if so, how did the team approach this issue? Throughout this section, it is unclear who was doing what, how many researchers were involved in data collection and data analysis, etc. Please provide clarifying detail.

**Thank you. We have added additional details and expanded the information sources of the methods section of the article (line 258-262):**

**“L.N undertook the capturing of the data for each project and regularly updated the project log, which was also supported by P.G. Once a project had secured funding and approved proposal, all the necessary information was added to the project log. Any changes made to a project afterwards, were updated in the project log. There was only an exception of one project which was excluded due to the funding that was not confirmed. Data for each project were continually captured and reviewed and sense checked monthly from the start of the ActEarly project (01.10.2019) to its completion (31.09.2024). Information was gathered through one-on-one meetings with researchers working within each ActEarly theme, and through executive meetings with senior academics and theme leaders. Each theme lead was asked to review the project list relevant to their theme, supplement the information given by the research fellows, and address any outstanding queries”.**

**This additional information has also been added to the mapping of projects section of the methods (line 310-312):**

**“The mapping of the projects and further analysis was undertaken by P.G, while P.G conducted the network analysis. M.B and L.N. L.P, J.S, S.I contributed to reviewing the analysis”.**

- The cover letter links to the protocol, but this is not referenced in the text; please include

**We have this statement within the methods section where the protocol is now referenced (line245-249):**

**“The cross-cutting evaluation theme of ActEarly, as noted in the protocol [18] focused on evaluating the programme as a whole, including exploring the potential impact of the programme on early life health, identifying the extent to which it fostered new collaborations and networks and built capacity, and the degree to which it influenced policy and practice” [18].**

Reviewer #1:

This is a very important evaluation that took a systems mapping approach to understand where the ActEarly programme projects took place within the wider child health system. It found that ActEarly demonstrated good coverage across the wider system and mostly met the priority areas identified by stakeholders.

As the authors highlight, a key strength of the approach is that it has the potential to be replicated in other settings to support the evaluation of large-scale system-level public health programmes.

I can agree that, as per criteria for publication:

1. The study presents the results of original research.

2. The results reported have not been published elsewhere.

3. Experiments, statistics, and other analyses are performed to a high technical standard and are described in sufficient detail.

4. Conclusions are presented in an appropriate fashion and are supported by the data.

5. The article is presented in an intelligible fashion and is written in standard English.

6. The research meets all applicable standards for the ethics of experimentation and research integrity.

7. The article adheres to appropriate reporting guidelines and community standards for data availability.

In order for the paper to be published I make the following suggestions for the authors:

Abstract

Line 41/42 - suggest clarify to “provided activities across the full child health system and corresponding early life core outcome indicators that were prioritised by relevant individuals, organisations and community groups”. Or, if this has been misinterpreted, rephrase to make clear what you are saying here.

**We would like to thank this reviewer for their positive and encouraging comments about the paper. We have responded to the comments in detail below, and in the paper.**

**We have clarified the statement in the abstract section as shown below (line42-23):**

**“We report on an approach to evaluate the extent to which a city-wide programme of interventions, delivered in two locations to improve child health (ActEarly) provided activities across the child health system and addressed early-life core outcomes which were selected based on priorities identified by key stakeholders.**

Line 44 – suggest a line to list examples of the ‘other information sources’ depending on word limit.

**The information sources have been detailed in the method section, due to the word count we are unable to include here.**

Line 47/48 – if space then mention the 3 outcome indicators that projects did not map onto, i.e. priority areas for stakeholders that have been missed by ActEarly.

**We have listed the three outcomes not mapped unto any ActEarly projects and the paragraph amended as shown below (58-59):**

**“Projects also mapped well onto 32 of the 35 outcomes across the COS domains, with the exception of Adult Obesity, Safety at Home, and Domestic Abuse. This suggests that ActEarly aligned well with the priority outcomes from local representatives, partners and community groups.”**

Introduction.

The authors provide a coherent background to the study, outlining the history of the project clearly and the aims of the present study.

Suggest subheadings to introduce the CHM and COS.

**The section has been divided into key headings and subheadings.**

Line 120- 125 – I would just question whether the ‘extent to which ActEarly reached its goal of enacting city-wide changes’ is the same as the extent to which ActEarly operates across different domains of a complex system of child health and wellbeing. Maybe for clarity here you could explain that the evaluation sought to explore the extent to which ActEarly met its aim of [x,y,z] through a systems evaluation which involved.

**Thank you for raising this point. The statement indicates that our evaluation sought to examine the extent to which ActEarly projects operated across the complex system of child health utilising the CHM and the outcomes prioritised by stakeholders. We have therefore amended the paragraph to read (line 141 - 143):**

**“Considering ActEarly overarching goal of enacting city-wide changes across the child health system, we were therefore interested in how ActEarly operated across this system utilising the Child health map and the Core outcomes set for early life”.**

Line 125 – provide example(s) of where the CHM has been used before and how effective it is.

**The CHM has not been used previously in the context of mapping projects as utilised in our research, but it has been referenced in other studies regarding the relevance of considering a whole systems approach to addressing child health inequalities in public health interventions. We have added the following statement below to the article (line 174-183):**

**“The Child Health Systems Map has been referenced in studies that emphasise the importance of systems thinking in addressing complex public health challenges. These works share a common recognition that understanding what works, and what does not, is essential for effective policy and programme evaluation. They argue that adopting a systems perspective supports the development of interventions that account for wider system influences, acknowledging that the factors underpinning health inequalities are inherently complex [22,23]. To the best of our knowledge, this study is the first to employ the child health system map by Jessiman and colleagues [7] as an approach for identifying areas of activity of a system-wide public health collaboration (ActEarly)”.**

Line 175-176 - suggest clarifying the sentence, does this mean older adult and childhood poverty are the highest in the country? Or in comparison to the national average? Suggest adding a couple of stats to clarify.

**Thank you for pointing this out. We have expanded the paragraph for clarity and added some statistics (lines 219-220 and 226-230).**

**“Around two thirds of the wards in Bradford had a higher proportion of relative child poverty than the national average of 19.9%”.**

**“About 47% of children live in poverty after housing costs in LBTH, substantially above the UK average of around 31% [28]. Across 20 local authority districts across England, 50% or more children and older people are reported as living in income deprived households. Tower Hamlets has the highest proportion at 71.3% for children and (61.1%) for adults”.**

Line 186 – I think this is supposed to be ‘Healthier Wealthier Families’

**Yes. This has been corrected.**

Line 212 – is Act Early the same age range as CHM? Suggest mentioning if it is/is not.

**We have included this information of the age focus of ActEarly to the methods section (line 275-276):**

**“Similar to CHM, ActEarly projects were focused on early years, school-aged children, and young adults”.**

Table 1 – are social environment and physical environment criteria the wrong way round (e.g. would assume open space, parks, green space etc would be physical environment) and family and social relationships etc would be social environment). Suggest double checking.

**We apologise for the error. This has been corrected.**

Results

Line 234 – suggest listing initials of ‘research team’ for clarity.

**The paragraph has been expanded to include the initials of research team (line 310-312):**

**“We mapped the information retrieved from each ActEarly project to domains within the CHM and the ActEarly COS (Table 1). The mapping of the projects and further analysis was undertaken by P.G, while P.G conducted the network analysis. M.B and L.N. L.P, J.S, and S.I contributed to reviewing of the analysis”.**

Line 297 – delete ‘is’:

**Deleted**

Line 298 – suggest use consistent language with Table 4 (e.g. rephrase to “activities related to ‘public health’ factors”):

**This has been rephrased to read (line 379-380):**

**“This highlights that activities related to public health factor within the child health map were most common across ActEarly”.**

Figure 1 – It’s a potentially effective and useful visual, however I suggest making sure each project name can be clearly seen as currently some areas of high density cannot be read.

**Thank you. A revised version of the figure has been uploaded. We will also release an interactive version and will provide a link for the paper before final publication.**

Discussion

Some discussion of the strengths and weaknesses of the CHM would be useful. Could be used in other contexts? How up-to-date is it? Are there domains that are missing or under or over-represented?

**Thank you. We have added more clarity and details to the areas mentioned in the discussion section. We have highlighted the context of the CHM which we used for our research, strengths and weakness including the domains that are missing (and how we addressed it in our revised map).**

**The paragraph below has been added to the introduction to highlight the context (164-172):**

**“Two local authority areas were selected to create locality-specific system maps, chosen for their contrasting geography, governance structures, and population profiles to inform the development of a generic map. Site 1 was an urban city in Northern England, while Site 2 was a rural county in the Southwest [7]. The sites differed in governance arrangements (unitary versus two-tier), levels of deprivation based on the Indices of Multiple Deprivation [19], and ethnic diversity [20]. Commonalities between the area-specific system maps, along with the removal of locality-specific factors, indicated that the CHM was applicable for use across English localities [7].**

**The paragraph below has been added to the discussion to further expand on other aspects (513-541):**

**“Jessiman et al. [7] anticipated that many of the factors and domains that were represented may not be exclusive to England alone, although this has yet to be tested or researched. The map can also be used to identify factors that may be influenced by local public health interventions and hence inform evaluation design” [7]. Within the context of a whole-systems approach to addressing child health inequalities, the CHM emerges as a relatively new framework designed to support strategies for reducing inequalities. In addition, we used the EY-COS [2], which facilitates the evaluation of individual interventions within system-change approaches. The EY-COS outcomes reflect priority areas identified at the start of ActEarly by system representatives, collaborating partners, and community members [2]. The EY-COS, applied in this study, enabled us to address aspects of the CHM domain that were not captured in the map. Examples include speech, language and communication, children get best start in life, child sedentary behaviour”.**

**“One of the strengths of the CHM lies in its value in demonstrating to stakeholders the need for policy approaches that address the broader systemic determinants of child health inequalities, extending beyond traditional public health interventions. In addition, the CHM also serves as a systems-thinking tool to assist Integrated Care Systems in recognising a broad spectrum of factors that contribute to child health inequalities [7]. Jessiman et al. [7] noted that the CHM is complex and potentially overwhelming, with participants suggesting the development of an interactive version to support easier interpretation and adaptation for use in local areas. This limitation was considered during our study; therefore, we engaged in a detailed analytical review of the existing CHM within each specific domain, examining all factors individually before adapting them for the revised ActEarly map. It was also noted that the CHM may also need more adequate information to understand the direction of influence between factors (as most factors are interlinked), hence during our analysis, we also considered the factors individually”.**

Reviewer #2: Thank you for the opportunity to review this paper. The approach taken by the authors is interesting and will make a great contribution to the literature. There are a few areas where the paper would benefit from more in-depth and critical engagement with its subject matter and the frameworks it has used. I list three cases that require particular attention below:

It would be good to see some more reflection on the systems map that was used, for example: for what purpose and in which context was this map developed? Who were the participants? And how might all three of these factors influence what is in- and excluded in the map? Then, you could reflect on how this links in with the current study. I.e. why was this map chosen? How does the purpose / context of the map link to that of ActEarly and are there some areas of focus for ActEarly that are not represented on the map?

**Again, we would like to thank this reviewer for such positive feedback on our approach. We have responded to the point about the context the map was developed as these was something that reviewer #1 also raised.**

**We have also edited the following paragraph in the introduction to expand further the research participants and purpose of the systems map (line 160-164, 147-152):**

**“Research participants included children and young people, their carers, and professionals from local authorities, the NHS, and CCGs, as well as representatives from third-sector organizations and elected members with expertise in child health from the two study areas. The final system map showing the determinants of child health inequalities was refined through input from experts in public health, local governance, and systems science” [7].**

**“The CHM was designed to better understand the complexity around child health inequalities and demonstrate how individual policies or interventions may or may not effectively impact on the various dynamic issues of child health [7]. The authors therefore sought to identify what factors drive child health and wellbeing in a local area and what factors drive child health inequalities between different groups of children and young people in a local area”.**

**We have also added the following paragraph in the introduction to expand further on why the map was chosen and how the purpose/context of the map link to that of ActEarly (line 152-156):**

**“ActEarly shares this systems-oriented perspective around child health inequalities, focusing on two local areas in England with high levels of child deprivation. Our research was to understand and highlight which of the factors that drive child health inequalities highlighted in the CHM were most addressed/targeted by ActEarly projects, thereby operationalising the CHM into practice within local contexts”.**

I would question the assertion that connectivity in the map equals significance of that part of the system to the system as a whole, as seems suggested in line 243. The systems map used was inevitably a product of a specific time, context and set of participants, and so which nodes are most connected in this map do therefore not necessarily mean they are of most significant in childhood health.

**Thank you for raising this important point. In our analysis, degree centrality was used as an indicator of how ActEarly activities are connected with the factors in the CHM, rather than as a measure of the causal importance. We have rephrased our statement as shown below within the network analysis section (line 326-328):**

**“Analysis calculated the degree centrality of ActEarly factors (activities) in the CHM (the number of connections between that factor and any other factors in the system) as this may provide an indication of the significance of that part of the system to the system as a whole, as well as the number of ActEarly projects operating within each CHM factor”.**

**In addition, we have also added this to our discussion to provide more clarity (580-588):**

**“In our research, degree centrality was used to indicate how connected ActEarly activities were with the factors in the CHM. Thus, we examined the distribution of ActEarly projects across CHM which allowed us to consider not only connectivity but also practical engagement (identifying which areas of the system were actively targeted by projects). Our research enabled us to highlight potential leverage points for system-level change, while acknowledging that significance in terms of health impact requires more evaluation. The systems map used in the study reflects a specific context, and participant set, and therefore the areas in our revised map with most ActEarly projects may not universally provide an indication of the most significant factors influencing child health inequality”.**

Discussion would expect some more critical reflection. In particular, I would like to have seen reflection on

- Are all projects that are reportedly on more social or economic aspects also more upstream in nature? I.e. do they not suffer from lifestyle drift where they may (for example) intend to target a households income status but do so by providing budget information.

**This has been added to our discussion(line 458-463):**

**“Considering the ActEarly revised map, we do not classify all projects addressing social or economic aspects of child health inequalities as upstream in nature. While some aimed to tackle structural determinants such as directly influencing income or housing, few addressed this through more immediate practical measures, for example, providing welfare advice. This illustrates the complexity of system-level change, where in some situations, a project may shift toward supporting individual behaviour”.**

- There are some factors in your table which are well-connected in the map but on which there are no interventions. In the discussion you write that these are more likely to be focused on adults or behaviour change. But some of the ones in your table include social contact, smoking, positive friendship groups, etc.

**Thank you. We do agree that not all the factors on the map that are well connected without ActEarly projects are linked to adults and behaviour change. In our discussion we stated that there are a couple of exceptions. We have amended the following paragraph in the discussion to provide more clarity.**

**“If we look at areas of the system where there were no ActEarly projects directly targeting a factor/s, it tended to be (with a couple of exceptions) where the focus was more on adults or older children, or those that link to behaviour change. In addition, some of these factors reside within the personal domain (for example social isolation, smoking) of the CHM and were included to illustrate how they have been linked to influencing other factors [7]. Due to this interlink between factors, further research is needed to understand how ActEarly project may have impacted on these areas of the CHM”.**

- You do briefly touch on that this method cannot show the impact that actions have had across the system. It would be good to expand on this either here or in the methods.

**We have edited the following paragraph in the discussion to expand this further (line 590-598):**

**“Care should also be taken to not over interpret the network, as there are no data that might indicate the strength of the impact on that area of the child health system by any individual project, nor are we able to say if there is any cumulative impact of projects either on one part of the system (any one node) or regions of the network as linked by relationships between the parts. It is merely indicative of ActEarly activity in that area. In addition, many projects operate across multiple domains or have indirect influence, which again highlights the complexity of interpreting connectivity and project activity as measures of impact. Our aim is to provide an overview of ActEarly reach and potential leverage points, while acknowledging that causal significance and system-wide impact require further evaluation”.**
